# Novel Technique of Endoscopic Ultrasonography for the Differential Diagnosis of Gallbladder Lesions and Intraductal Papillary Mucinous Neoplasms: A Single-Center Prospective Study

**DOI:** 10.3390/diagnostics13132132

**Published:** 2023-06-21

**Authors:** Yasunobu Yamashita, Reiko Ashida, Takaaki Tamura, Toshio Shimokawa, Hirofumi Yamazaki, Yuki Kawaji, Takashi Tamura, Keiichi Hatamaru, Masahiro Itonaga, Masayuki Kitano

**Affiliations:** 1 Second Department of Internal Medicine, Wakayama Medical University, 811-1, Kimiidera, Wakayama 641-0012, Japan; rashida@wakayama-med.ac.jp (R.A.); tamutoko@wakayama-med.ac.jp (T.T.); hirofumi.y.nagoya@gmail.com (H.Y.); y.kawaji1985@gmail.com (Y.K.); ttakashi@wakayama-med.ac.jp (T.T.); papepo51@wakayama-med.ac.jp (K.H.); masaitonaga0907@gmail.com (M.I.); kitano@wakayama-med.ac.jp (M.K.); 2 Department of Human Pathology, Wakayama Medical University, 811-1, Kimiidera, Wakayama 641-0012, Japan; 3 Clinical Study Support Center, Wakayama Medical University Hospital, 811-1, Kimiidera, Wakayama 641-0012, Japan; toshibow2000@gmail.com

**Keywords:** detective flow imaging endoscopic ultrasonography, Doppler endoscopic ultrasonography, novel technique, gallbladder cancer, intraductal papillary mucinous neoplasm

## Abstract

Detective flow imaging endoscopic ultrasonography (DFI-EUS) is an innovative imaging modality that was developed to detect fine vessels and low-velocity blood flow without contrast agents. We evaluate its utility for the differential diagnosis of gallbladder lesions and intraductal papillary mucinous neoplasms (IPMNs). We enrolled patients who underwent DFI-EUS, e-FLOW EUS, and contrast-enhanced EUS for gallbladder lesions or IPMNs. The detection of vessels using DFI-EUS and e-FLOW EUS was compared with that via contrast-enhanced EUS and pathological findings. The vessel pattern was also categorized as regular or irregular. Of the 33 lesions included, there were final diagnoses of 13 IPMNs and 20 gallbladder lesions. DFI-EUS was significantly superior to e-FLOW EUS for discriminating between mural nodules and mucous clots and between solid gallbladder lesions and sludge using the presence or absence of vessel detection in lesions (*p* = 0.005). An irregular vessel pattern with DFI-EUS was a significant predictor of malignant gallbladder lesions (*p* = 0.002). DFI-EUS is more sensitive than e-FLOW-EUS for vessel detection and the differential diagnosis of gallbladder lesions and IPMNs. Vessel evaluation using DFI-EUS may be a useful and simple method for differentiating between mural nodules and mucous clots in IPMN, between solid gallbladder lesions and sludge, and between malignant and benign gallbladder lesions.

## 1. Introduction

Pancreatobiliary diseases are important causes of morbidity and mortality worldwide. Among the imaging modalities, transabdominal ultrasonography is the most non-invasive, cheapest and widely used method. However, it has some limitations. When performing transabdominal ultrasonography imaging, the intervention of gas, bone and fat can create problems. Endoscopic ultrasonography (EUS) was developed as a solution to these issues. EUS is the most reliable and efficient diagnostic modality for pancreatobiliary disease owing to its high spatial resolution, which allows for the detection of small lesions in pancreatobiliary disease. There is difficulty in making differential diagnoses based on EUS imaging characteristics alone, however, because the majority of lesions are hypoechoic. The assessment of vascularity is another approach that can be used for differential diagnosis. Doppler imaging modalities, such as color-Doppler EUS, power Doppler EUS, and e-FLOW EUS, are used in real-time checking of vascularity. Of these methods, e-FLOW EUS is a directional power Doppler ultrasonography method that has better spatial and temporal resolutions than the others. However, conventional EUS Doppler modes, including e-FLOW EUS, are not suited to the visualization of fine vessels and slow flow. On the other hand, contrast-enhanced EUS (CE-EUS) is better for detecting blood flow and does not have some of these limitations. Assessments of the utility of CE-EUS for detecting intraductal papillary mucinous neoplasms (IPMNs) and gallbladder lesions have been reported in many articles [1,2,3,4,5,6,7,8,9,10,11]. Moreover, CE-EUS is increasingly being used for the differential diagnosis of pancreatobiliary lesions, offering advantages over contrast-enhanced computed tomography (CT) and magnetic resonance imaging (MRI) in patients with contraindications to these modalities, such as those with renal failure or contrast allergy. CE-EUS also allows for dynamic and repeat examinations. However, the use of contrast agents means that there are some caveats concerning its use, and recent advances in technology have led to the development of detective flow imaging EUS (DFI-EUS) to overcome the problems associated with conventional Doppler-mode EUS. DFI-EUS is superior to CE-EUS for the following reasons. The assessment with DFI-EUS requires no relevant extra time and cost for the administration of contrast agents compared to routine EUS because it is necessary to push only one DFI button without using contrast agents and special equipment. However, at this time, DFI-EUS can only be done using convex-type endoscopes (GF-UCT260; Olympus, Tokyo, Japan) with ultrasound observation systems (ARIETTA 850; FUJIFILM Healthcare, Tokyo, Japan) because DFI-EUS is a newly development technique. DFI-EUS is not yet widespread. Therefore, few reports have examined its clinical utility because the method is so new [12,13]. In this study, we assess the use of DFI-EUS for vessel detection and the differential diagnosis of IPMNs and gallbladder lesions by a comparison of the results with those of e-FLOW EUS for CE-EUS and pathological diagnosis.

## 2. Materials and Methods

### 2.1. Patients

Between January 2020 and December 2021, 58 patients who underwent DFI-EUS, e-FLOW EUS, and CE-EUS at Wakayama Medical University Hospital for gallbladder lesions or IPMNs detected by CT, MRI, and/or transabdominal ultrasonography were prospectively enrolled.

**Figure 1:**
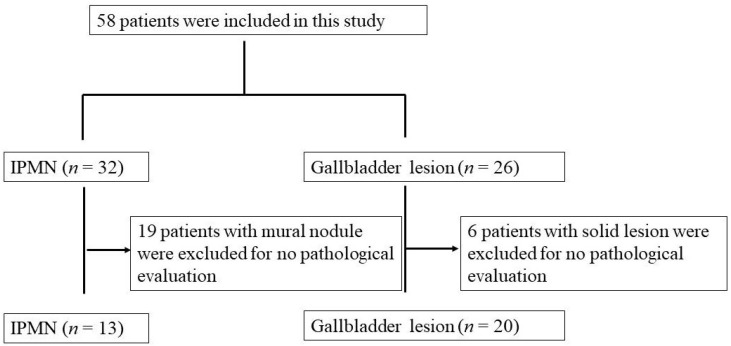
Flowchart of study selection.

The inclusion criteria for this study were: age >20 years, and DFI-EUS, e-FLOW EUS, and CE-EUS imaging all being performed on the same day. CE-EUS was done at the end of examination because it affected DFI-EUS and e-FLOW EUS images. Patients suspected of having gallbladder sludge or mucous clots in the IPMN after CE-EUS were followed-up for at least 1 year to confirm an absence of change. Patients with a mural nodule in IPMN underwent surgical resection for diagnosis. Patients with a solid gallbladder lesion underwent surgical resection or EUS-guided fine needle aspiration (EUS-FNA) for diagnosis. Finally, 33 patients were included in this study according to inclusion criteria as surgical resection was not performed for 19 patients with mural nodules in IPMN and 6 patients with solid gallbladder lesions (Figure 1). There were 19 men and 14 women, and the median age was 67.3 ± 11.4 years. Blood vessels and vessel patterns were assessed, with vessel patterns being categorized as regular or irregular.

This study was approved by the Wakayama Medical University Ethics Committee (Nos. 2492 and 2496), and all patients provided informed consent.

### 2.2. Study Design

This study evaluated the utility of DFI-EUS for the differential diagnosis of IPMN and gallbladder lesions. The primary aim was to measure the accuracy of DFI-EUS for differentiating between mural nodules and mucous clots in IPMN, and to differentiate between solid gallbladder lesions and sludge. A lesion with vessels was defined as a mural nodule or a solid gallbladder lesion, and a lesion without vessels was defined as a mucous clot or sludge. The accuracy of DFI-EUS in the differential diagnoses was compared with that of e-FLOW EUS. For mucous clots and sludge, the absence of blood flow was confirmed by CE-EUS. A secondary outcome measure was the ability to differentiate between malignant and benign variations of IPMN and gallbladder lesions. A lesion with a regular vessel pattern was defined as benign, and a lesion with an irregular vessel pattern was defined as malignant.

### 2.3. EUS Procedure

**Figure 2:**
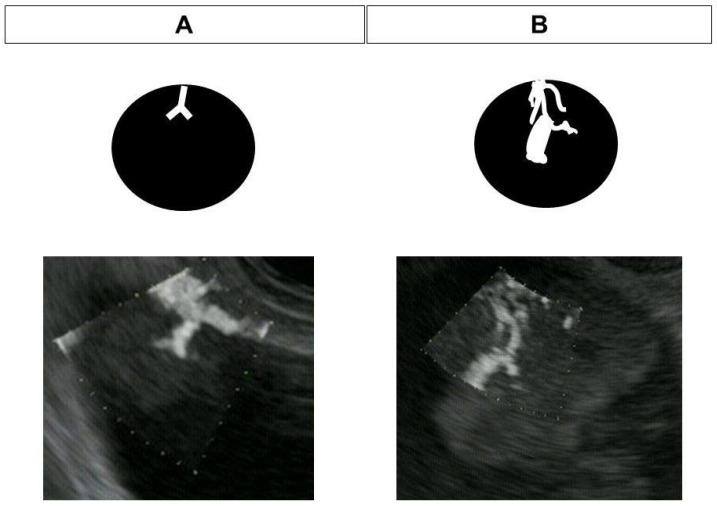
Typical images of regular and irregular vessel patterns (**A**) Regular vessels. (**B**) Irregular vessels.

Convex-type endoscopes (GF-UCT260; Olympus, Tokyo, Japan) with ultrasound observation systems (ARIETTA 850; FUJIFILM Healthcare, Tokyo, Japan) were used. The patient was placed in the left lateral position under diazepam-induced sedation for EUS, and heart rate, saturation, and blood pressure were monitored. The color gain, dynamic range, and transmission frequency were set to 65, 83, and 5.0 MHz, respectively, for DFI-EUS, and 52, 83, and 5.0 MHz for e-FLOW EUS. The color gain settings were optimized before starting the study to minimize the noise in lesions in either mode, and these values were then fixed across all patients. EUS images were recorded for the subsequent evaluation of vascularity. Assessment of vessels was performed on the recorded EUS images by three endosonographers who had at least 10 years of EUS experience, and who were blinded to all other imaging and patient information. Vessels were assessed as absent/present or regular/irregular vessels. The terms regular vessel images refers to smooth and linear vessels in the tumor, while irregular vessels are defined as tortuous vessels with a caliber change (Figure 2).

### 2.4. DFI-EUS

**Figure 3:**
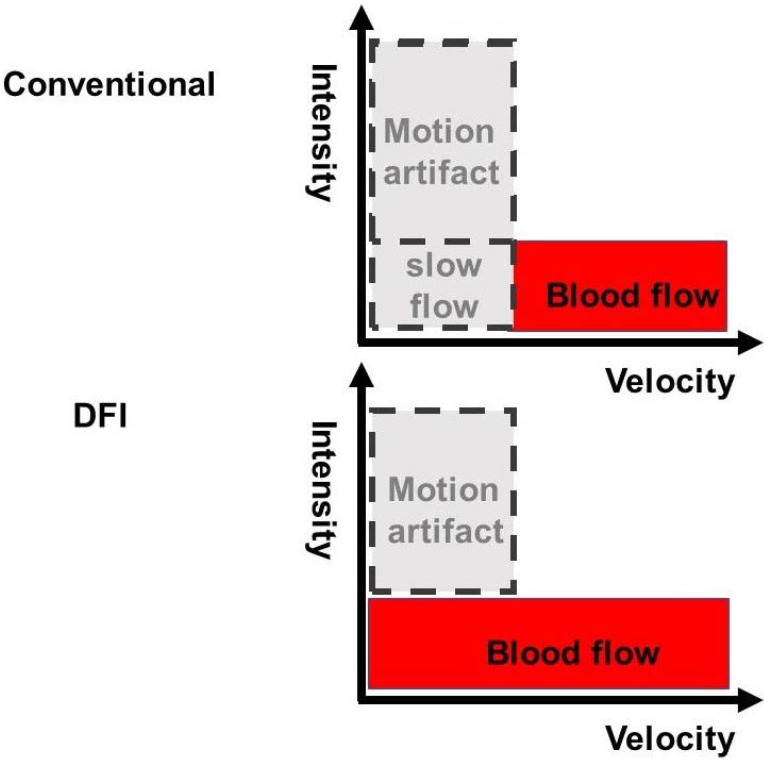
Conventional Doppler and DFI images. Conventional Doppler techniques use a single-dimension wall filter to remove motion artifacts, resulting in the loss of the slow component. This suppression of the slow component results in data loss and the subsequent lost visibility of flow in smaller vessels. Instead of suppressing these low-flow signals, DFI separates them from overlying tissue motion artifacts while simultaneously preserving low-flow components and providing detail and definition.

DFI-EUS, an innovative Doppler US technique, can detect very slow flow and microvascular vessels, doing so without motion artifacts or the need for contrast agents. In terms of Doppler ultrasonography, the ultrasonic Doppler signals represent not only blood flow, but also motion artifacts. Therefore, the signals from the motion artifact overlap with low-speed flow components (Figure 3). The slow component is then largely lost because conventional Doppler techniques apply a single-dimensional wall filter to remove motion artifacts. The suppression of slow components results in loss of data, and the subsequent loss of visible flow in smaller vessels. Rather than suppressing these low-flow signals, DFI-EUS separates them from overlying tissue motion artifacts, allowing their detection (Figure 3). Conventional Doppler techniques were predominantly developed with the goal of visualizing blood flow at high resolutions, and the e-FLOW technique currently has the best resolution. Moving beyond the accomplishments of conventional Doppler techniques, DFI-EUS can visualize lower-velocity blood flow with high resolution. DFI-EUS thus expands the range of blood flow that can be visualized and allows for the visualization of low microvascular flow, which cannot be seen using other methods.

### 2.5. Contrast-Enhanced EUS

The CE-EUS used Perflubutane (Sonazoid^®^ GE Healthcare Pharm, Tokyo, Japan), which is a second-generation ultrasonography contrast agent composed of perfluorobutane microbubbles with a median diameter of 2–3 μm. After reconstitution with 2 mL of sterile water for injection, 0.7 mL of the agent was administered through a peripheral vein. For CH-EUS, the extended pure harmonic detection method was used with the mechanical index set at 0.3.

### 2.6. Statistical Analysis

The JMP Pro version 13 (SAS Institute Inc., Cary, NC, USA) was used for statical analysis. The comparison of vessel detection between DFI-EUS and e-FLOW EUS was performed with McNemar’s test. Inter-rater agreement was evaluated with Landis and Koch proposals (Fleiss’ kappa value of <0: no agreement; 0–0.20, slight agreement; 0.21–0.40, fair agreement; 0.41–0.60, moderate agreement; 0.61–0.80, substantial agreement; and 0.81–1, high agreement). The qualitative variables were used to compare categorical variables with Fisher’s exact test. A *p* value less than 0.05 is statistically significant.

## 3. Results

**Figure 4:**
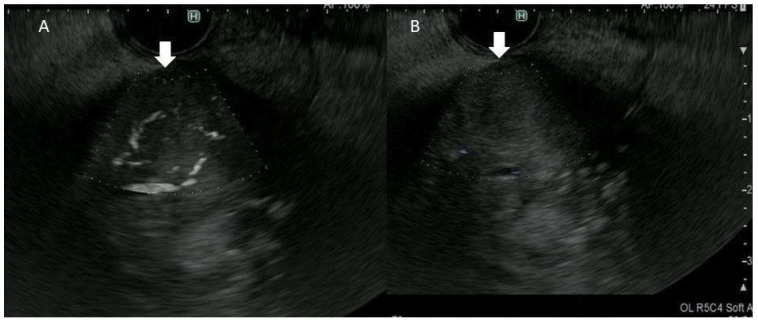
The difference between detective flow imaging (DFI) and e-FLOW endoscopic ultrasonography for vessel detection in malignant gallbladder lesion. (**A**) DFI-endoscopic ultrasonography: the vessels were depicted in the gallbladder lesion (arrow). (**B**) e-FLOW endoscopic ultrasonography: the vessels were not depicted in gallbladder lesion (arrow).

**Figure 5:**
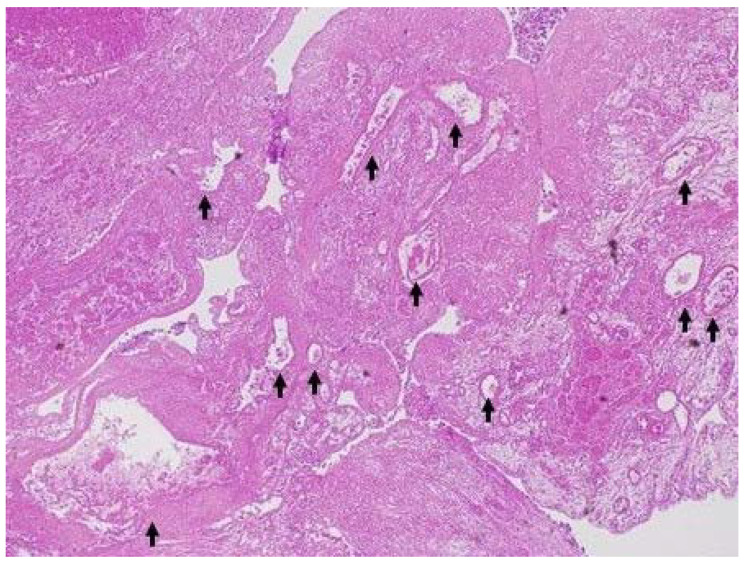
**A representative case of resected malignant gallbladder lesion.** Resected specimen showed moderately differentiated adenocarcinoma of gallbladder. The irregular vessels (arrow) were detected in the lesion (H&E, ×40).

**Figure 6:**
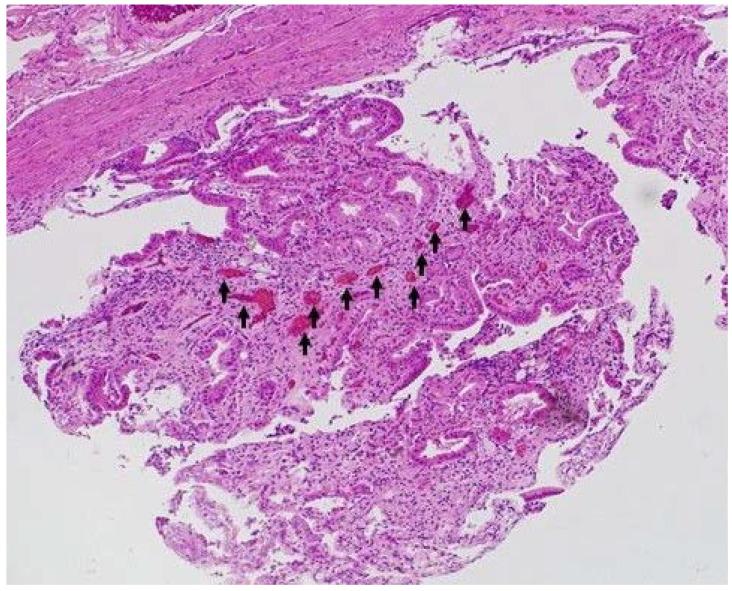
**A representative case of resected benign gallbladder lesion.** Resected specimen showed pyloric gland adenoma of gallbladder. The relatively regular vessels (arrow) were detected in the lesion (H&E, ×40).

The patient characteristics are shown in Table 1. The final diagnoses of 33 examined lesions were IPMN in 13 patients and gallbladder lesion in 20 patients. When IPMNs were assessed using CE-EUS, four were categorized as mural nodules and nine as mucous clots. In accordance with IPMN guidelines [5], surgical resection was performed for mural nodules (median, 8.5 mm; range, 5–24). IPMNs with mural nodules were categorized in accordance with the UICC TNM classification as intraductal papillary mucinous adenoma (IPMA) (*n* = 1) or intraductal papillary mucinous carcinoma (IPMC) (*n* = 3; stage 0, *n* = 1 and IA, *n* = 2). The assessment of the differences in vessel type (regular/irregular vessel) between malignant and benign IPMN was not performed because there were only four surgical IPMN lesions, including one benign IPMN. The examination of gallbladder lesions by CE-EUS led to a diagnosis of gallbladder sludge in 8 cases and solid lesions in 12 cases. Histopathological examination of the solid gallbladder lesions with EUS-FNA (*n* = 5) or surgery (*n* = 7) revealed carcinoma (*n* = 8), metastasis of renal cancer (*n* = 1), adenoma (*n* = 1), cholesterol polyp (*n* = 1), and chronic cholecystitis (*n* = 1). Inter-rater agreement regarding the assessment of vascularity was high (kappa value = 0.88). The accuracies of DFI-EUS and e-FLOW-EUS for differentiating mural nodules from mucous clots in IPMN were 100% (13/13) and 77% (10/13), respectively. The accuracies of DFI-EUS and e-FLOW EUS for discriminating between solid lesions and sludge in gallbladder lesions were 95% (19/20) and 70% (14/20), respectively. DFI-EUS (32/33, 97%) was thus significantly superior to e-FLOW EUS in discriminating between mural nodules and mucous clots in IPMN, and also between solid gallbladder lesions and gallbladder sludge (24/33, 73%) (*p* = 0.005). In 9 of 33 cases (27%), lesions were not adequately diagnosed on e-FLOW EUS. The median size of lesions missed on e-FLOW EUS was 15 mm (range, 5–35 mm). We find it particularly concerning that gallbladder cancer, measuring 35 mm in size, was missed on e-FLOW EUS (Figure 4). Irregular vessel patterns were detected in eight of the nine malignant gallbladder lesions (89%), and a regular pattern was detected in all three benign lesions (100%), with the rate of irregular vessels being significantly higher in malignant lesions than in benign lesions (*p* = 0.002) (Table 2). DFI-EUS was able to diagnose all benign gallbladder lesions > 10 mm in diameter via surgical resection. Inter-rater agreement regarding the assessment of vessel type (regular/irregular vessel) was substantial (kappa value = 0.63). Comparison with pathological findings of surgical specimens revealed that the irregular vessels were detected in the malignant lesions and the relatively regular vessels were detected in the benign lesions (Figure 5 and Figure 6). The sensitivity, specificity, and accuracy for detection of malignant gallbladder lesions with DFI-EUS were 89% (8/9), 100% (3/3), and 92% (11/12), respectively (Table 2).

## 4. Discussion

According IPMN guidelines, EUS plays an important role in addressing worrisome features including cyst ≥3 cm, thickened and enhanced cyst wall, mural nodule < 5 mm, a main pancreatic duct 5–9 mm, lymphadenopathy, abrupt changes in the caliber of the pancreatic duct with distal pancreatic atrophy, cyst growth rate ≥5 mm in two years, and elevated CA 19-9 [14]. In particular, identifying mural nodules in IPMN is important because the differential diagnosis between mural nodules ≥5 mm at high risk of stigmata and mucous clots is a very notable factor in decisions on surgical intervention (IPMN guidelines, 2017) [14]. Diagnosis of gallbladder lesions is also important because gallbladder lesions >10 mm in diameter should be resected because they have the potential for malignancy [15]. Moreover, imaging modalities plays an important role in the preoperative diagnosis of IPMN and gallbladder lesions because preoperative pathological evaluation is difficult. However, discrimination between mural nodules and mucous clots and between solid gallbladder lesions and sludge is sometimes difficult. Vascular assessment by Doppler imaging is used for such discrimination, but its ability to detect the fine vessels is limited, and there can be blooming and overpainting artifacts. In these situations, CE-EUS is widely used in clinical practice because it is more useful for this discrimination than Doppler EUS [1,16]. However, the contrast agent used for CE-EUS is relatively expensive (approximately $150 per procedure), intravenous injection is required, and additional special equipment is needed. There has therefore been a need for a Doppler imaging method that enables the detection of fine vessels with the same resolution and sensitivity as CE-EUS, but without the need for a contrast agent; this led to the development of DFI-EUS.

In this study, we evaluated the vascularity of IPMN and gallbladder lesions using DFI-EUS. In 32 of 33 lesions, DFI-EUS facilitated correct discrimination between mural nodules and mucous clots and between solid gallbladder lesions and gallbladder sludge. By contrast, e-FLOW EUS, which has the best resolution among the three conventional Doppler modes, missed diagnosis in 9 of 33 cases (27%). DFI-EUS was therefore found to be significantly superior to e-FLOW EUS in the differential diagnosis in IPMN and gallbladder lesions. We found it particularly concerning that gallbladder cancer measuring 35 mm in size was missed on e-FLOW EUS. A reason for detection with e-FLOW-EUS being difficult in advanced cancer could be because the blood flow in advanced cancers with neovascular attributes is slow or often intermittent [17]. DFI-EUS is therefore recommended for the assessment of vascularity and offers a viable alternative to CE-EUS for the assessment of vascularity without the need for a contrast agent. To some extent, it is possible to evaluate the discrimination between mural nodules and mucous clots and between solid gallbladder lesions and gallbladder sludge with B-mode-EUS only. However, the judgement is subjective, and so proficiency in evaluation influences results. DFI-EUS is therefore superior to the B-mode version because of its ability to perform a comparatively simple and objective assessment by determining the presence or absence of vessels without contrast agent and special equipment.

We also evaluated the effectiveness of DFI-EUS for discriminating between malignant and benign gallbladder lesions (this was not done for IPMN because only four surgical IPMN lesions, including one benign IPMN, were resected). Irregular vessel patterns were detected in 89% of malignant gallbladder lesions, and regular vessel patterns were detected in 100% of benign lesions. An irregular vessel pattern was therefore found to be a significant predictor of malignancy in gallbladder lesions. Unnecessary surgical resection was performed for three benign gallbladder lesions in this study because gallbladder lesions >10 mm in diameter had the potential for malignancy. On the other hand, it was possible to diagnose gallbladder lesions as benign in all cases with DFI-EUS. In the previous report, the sensitivity, specificity, and accuracy were 77%, 88%, and 86%, respectively, when the lesion size classification (gallbladder lesions >10 mm in diameter) was defined as a malignant gallbladder lesion [2]. In terms of DFI-EUS, the sensitivity, specificity, and accuracy for detecting malignant gallbladder lesions with DFI-EUS were 89%, 100%, and 92%, respectively. Although the number of cases for a previous study was 125 and only 20 for our study, which is a limitation in terms of the number of cases, the use of DFI-EUS may enable more accurate preoperative diagnosis than one performed using lesion size. In a previous report, the sensitivity, specificity, and accuracy of measurements for malignant gallbladder lesions with CE-EUS were 90.3%, 96.6%, and 94.4%, respectively, when a malignant gallbladder lesion was defined as a lesion with an irregular vessel (same definition in this study) [3]. Although the number of cases for the previous study was 93 and that for our study was 20, which is a limitation in terms of the number of cases, the diagnostic ability for malignant gallbladder with CE-EUS was the same as that in this study. Therefore, DFI-EUS has the same high diagnostic performance as CE-EUS. Moreover, the use of DFI-EUS without a contrast agent and special equipment may be a viable alternative method to CE-EUS. To the best of our knowledge, this is the first report demonstrating the usefulness of DFI-EUS for the differential diagnosis of gallbladder lesions. Pathological findings revealed that malignant gallbladder lesions have more irregular vessels than benign gallbladder lesions. Bergers et al. reported the morphological characteristics of malignant tumor vessels as being tortuous, dilated, or irregular [17]. Moreover, the examination of vessels in esophageal cancer using narrow-band imaging (NBI), which improves the visibility of blood vessels, found that it could predict malignancy, and NBI is now widely for the diagnosis of esophageal cancer in clinical practice [18,19]. In the determination of malignancy in cases of gallbladder cancer, DFI-EUS may prove to be as important as NBI is for esophageal cancer.

We also examined the optimal settings for Doppler imaging including DFI-EUS and e-FLOW EUS. When the color gain is too low, the detection of vessels is difficult. However, the stronger the vessel signal becomes, the stronger noise only occurs without detecting the other vessels with a slower flow [20]. In this study, we determined the optimal color gain settings for DFI-EUS and e-FLOW EUS so that noise within lesions was minimized, and the color gain was then fixed across all patients to prevent the color gain settings from affecting the results. However, color gain does not necessarily affect the sensitivity for detecting vessels, but rather the strength of signals in vessels within a certain range of color gain [20].

It is important to compare the results of DFI-EUS with those obtained via transabdominal ultrasonography, another microvascular imaging technique. Diagnostic studies on various diseases have shown that the utility of transabdominal ultrasonography for microvascular imaging is similar to that of DFI [21,22,23,24,25,26,27,28,29]. Super microvascular imaging (SMI), another microvascular imaging technique, provides significantly more information than conventional Doppler imaging with respect to vascularity and is useful for the differential diagnosis of thyroid lesions, breast lesions, liver lesions, renal lesions, and cervical lymph nodes [21,22,23,24,25,26,27,28,29]. However, there are no reports on its utility for pancreatic lesions. There is only one report on the utility of SMI as a microvascular imaging technique for gallbladder lesions. In this study, which Kin et al. reported that a change in the caliber of vessels significantly predicted malignant gallbladder lesions [30]. This result is consistent with that of this study. When compared with SMI using transabdominal US, it can be seen that DFI-EUS has some advantages, particularly regarding the difficulty of using transabdominal US to perform microvascular imaging in obese individuals, where the depth of subcutaneous fat affects the clarity of the imaging [31]. Microvascular imaging using EUS (DFI-EUS) may therefore be superior to microvascular imaging using transabdominal ultrasonography.

This study has three main limitations. First, it is a single-center study with a small study population, meaning that further studies of DFI-EUS with larger cohorts from multiple centers are needed in the future. Second, sludge and mucous clots were confirmed by CE-EUS rather than by pathological analysis. However, it is difficult to perform surgical resection because in clinical practice, mucous clots and gallbladder sludge are evaluated by CE-EUS and then followed-up. In these cases, we confirmed that there was no change after one year. Third, we fixed the optimal color gains to compare both modes under the same conditions; however, the optimal color gains for both modes differed from case to case.

## 5. Conclusions

DFI-EUS was found to be more sensitive than e-FLOW-EUS for vessel detection and the differential diagnosis of gallbladder lesions and IPMN. We determined that an irregular vessel pattern with DFI-EUS also predicts malignant gallbladder lesions. The evaluation of vessels using DFI-EUS may be a useful and simple method for differentiating between mural nodules and mucous clots in IPMN, between solid gallbladder lesions and gallbladder sludge, and between malignant and benign gallbladder lesions.

## Figures and Tables

**Table 1:** Characteristics of Patients with IPMN and Gallbladder Lesions.

	IPMN (*n* = 13)	Gallbladder Lesion (*n* = 20)
Age, years, mean ± SD	66.9 ± 13.0	67.6 ± 10.5
Sex, male/female	6/7	13/7
Final diagnosis	mucous clot (9), mural nodule (4)	sludge (8), solid lesion (12)
Mural nodule or solid gallbladder lesion size, median (range)	8.5 (5–24) mm	26 (10–44) mm
Final diagnosis of mural nodule or solid gallbladder lesion	IPMA (1), IPMC (3); stage 0 (1), stage IA (2)	carcinoma (8), metastasis of renal cancer (1), adenoma (1), cholesterol polyp (1), chronic cholecystitis (1)

IPMN, intraductal papillary mucinous neoplasm; SD, standard deviation; IPMA, intraductal papillary mucinous adenoma; IPMC, intraductal papillary mucinous carcinoma.

**Table 2:** Vessel Patterns on DFI-EUS and Malignancy (benign or malignant) in Solid Gallbladder Lesions.

	Final Diagnosis (Pathology)	
	Benign (*n* = 3)	Malignant (*n* = 9)
DFI-EUS finding		
Regular vessel	3 (100%)	1 (11%)
Irregular vessel	0 (0%)	8 (89%)

*p* = 0.002 DFI-EUS, detective flow imaging endoscopic ultrasonography.

## Data Availability

The raw data are available with the consent of the corresponding author.
